# Feasibility of a Machine Learning-Based Smartphone Application in Detecting Depression and Anxiety in a Generally Senior Population

**DOI:** 10.3389/fpsyg.2022.811517

**Published:** 2022-04-08

**Authors:** David Lin, Tahmida Nazreen, Tomasz Rutowski, Yang Lu, Amir Harati, Elizabeth Shriberg, Piotr Chlebek, Michael Aratow

**Affiliations:** Ellipsis Health, San Francisco, CA, United States

**Keywords:** mental health screening, machine learning, smartphone, speech, NLP, artificial intelligence, behavioral health monitoring, biomarkers

## Abstract

**Background:**

Depression and anxiety create a large health burden and increase the risk of premature mortality. Mental health screening is vital, but more sophisticated screening and monitoring methods are needed. The Ellipsis Health App addresses this need by using semantic information from recorded speech to screen for depression and anxiety.

**Objectives:**

The primary aim of this study is to determine the feasibility of collecting weekly voice samples for mental health screening. Additionally, we aim to demonstrate portability and improved performance of Ellipsis’ machine learning models for patients of various ages.

**Methods:**

Study participants were current patients at Desert Oasis Healthcare, mean age 63 years (SD = 10.3). Two non-randomized cohorts participated: one with a documented history of depression within 24 months prior to the study (Group Positive), and the other without depression (Group Negative). Participants recorded 5-min voice samples weekly for 6 weeks *via* the Ellipsis Health App. They also completed PHQ-8 and GAD-7 questionnaires to assess for depression and anxiety, respectively.

**Results:**

Protocol completion rate was 61% for both groups. Use beyond protocol was 27% for Group Positive and 9% for Group Negative. The Ellipsis Health App showed an AUC of 0.82 for the combined groups when compared to the PHQ-8 and GAD-7 with a threshold score of 10. Performance was high for senior participants as well as younger age ranges. Additionally, many participants spoke longer than the required 5 min.

**Conclusion:**

The Ellipsis Health App demonstrated feasibility in using voice recordings to screen for depression and anxiety among various age groups and the machine learning models using Transformer methodology maintain performance and improve over LSTM methodology when applied to the study population.

## Introduction

Depression and anxiety are among the most prevalent mental health disorders internationally and in the United States. A national survey revealed that 8.1% of American adults of ages 20 and over had depression in a given 2-week period (Brody et al., 2018). Other surveys estimate a 3.1% prevalence rate of generalized anxiety disorders (GAD) present in the US population (Stein and Sareen, 2015). Depression and anxiety often co-occur, impacting the global economy by $1 trillion yearly (World Health Organization, 2021) and affecting over 280 million people worldwide [Institute for Health Metrics and Evaluation (IHME), 2019]. They are also highly associated with mental health-related disease burden, which in turn leads to higher risk of premature mortality (Chiu et al., 2018).

The high prevalence and debilitating consequences of depression and anxiety highlight the importance of screening across the life spectrum (Siu et al., 2016). Without screening, only 50% of depressed patients are identified (Akincigil and Matthews, 2017). Current screening methods such as the Patient Health Questionnaire-9 (PHQ-9) for depression (Löwe et al., 2004) and the Generalized Anxiety Disorder-7 (GAD-7) for anxiety (Spitzer et al., 2006) have limitations (Staples et al., 2019) including possible inflation of scores for gender and sexual minorities (Borgogna et al., 2021). Literacy level and/or visual impairments can require administration by a member of the care team, increasing time to completion. Additionally, traditional paper-and-pencil questionnaires also lack engagement, and scores can be inflated, exacerbating non-compliance and inadequate treatment (Demmin and Silverstein, 2020). Therefore, a more efficient and convenient way to screen and monitor depression and anxiety is needed.

### Mental Health Screening Using Technology

Smartphone audio can be easily recorded and transmitted, allowing accessible screening and monitoring of mental health. Audio-only capture is easier than video, especially with advancements in microphone smartphone technology (Cohut, 2020), and video requiring more effort on the part of the user for adequate facial positioning. It follows that an initial, informed digital approach to mental health screening utilizes machine learning for audio data analysis to improve prediction accuracy, a high-value research area for future digital solutions.

There is extensive research on detecting anxiety and depression through acoustic and semantic aspects of speech using machine learning techniques (Cho et al., 2019; Low et al., 2020). Experiments on speech acoustics have created models *via* feature extraction (Cohn et al., 2018; Stasak et al., 2019). Newer approaches apply deep learning techniques and show improved performance (Ringeval et al., 2019; Zhao et al., 2020). In ongoing work, large corpora of transcribed speech are used for natural language processing (NLP) training to further develop semantic speech analysis (Lan et al., 2019; Yang et al., 2019). The most relevant NLP advancements have been used for transfer learning and improvements in deep learning architecture like transformers (Bengio, 2012; Vaswani et al., 2017), which maintain performance without using prohibitive amounts of labeled data (Rutowski et al., 2020; Harati et al., 2021).

Many mental health apps are available, but user engagement and clinical adoption have proven challenging (Rasche et al., 2018; Bauer et al., 2020). Some studies have begun to propose methods for determining indicators of user engagement for these types of apps. Apps that incorporate one or more techniques such as mindfulness/meditation, peer support and trackers have significantly higher daily open rates, daily minutes of use and 30-day user retention than apps that focus on breathing exercise or psychoeducation. Daily use patterns show that usage peaked during evening hours for apps that incorporate tracker, psychoeducation and peer support, whereas usage was high during morning and nighttime for mindfulness/meditation apps (Baumel et al., 2019; Ng et al., 2019). Few existing technologies require a sample of the user’s speech; it is therefore unknown how participants would react to speech sample analysis for mental health.

Because speech is unique to an individual, people are rightfully concerned about speech capture, which could not only reveal their identity but their personal health information. In a healthcare environment, compliance with the Health Insurance Portability and Accountability Act (HIPAA; United States Department of Health and Human Services (HHS), 2021) guidelines ensures data privacy and security, but many digital health tools fall outside of this scenario and therefore not bound by HIPAA. However, users should be informed of their data that is collected and possibly shared. Best practices include treating this data as if it were covered by HIPAA, providing clear and transparent terms of use and consents, and prohibition of secondary selling or sharing the data for any purposes other than to directly support the function of the digital health tool. Ellipsis Health follows these best practices, and as many other machine learning/AI companies, does not listen or view all the data collected (which could number in the millions), as this data is used in aggregate to train the algorithms created by the company. Only in cases of troubleshooting are individual voices or data viewed, and in the case of speech, cannot be mapped to a particular user. Additionally, users should always be able to request their data to be deleted if they are uncomfortable with a company’s privacy and security practices.

**Figure 1:**
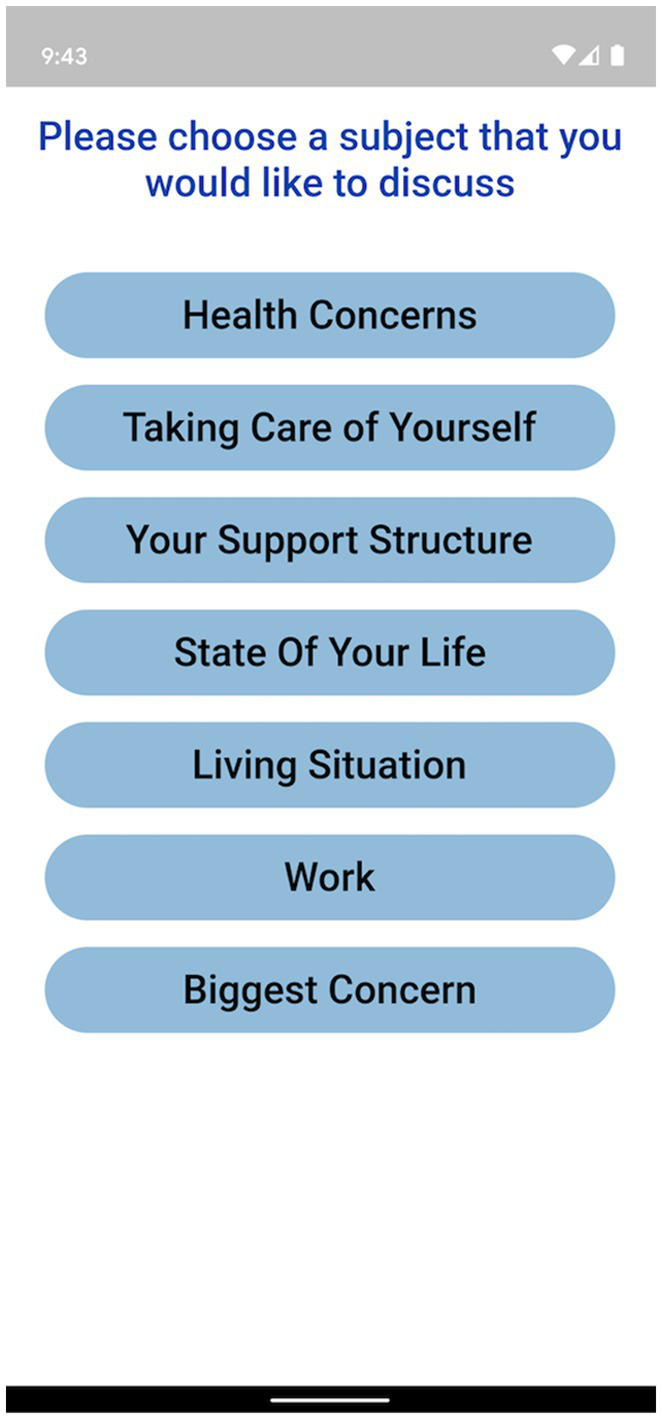
Topic choice for Ellipsis Health App participants.

**Figure 2:**
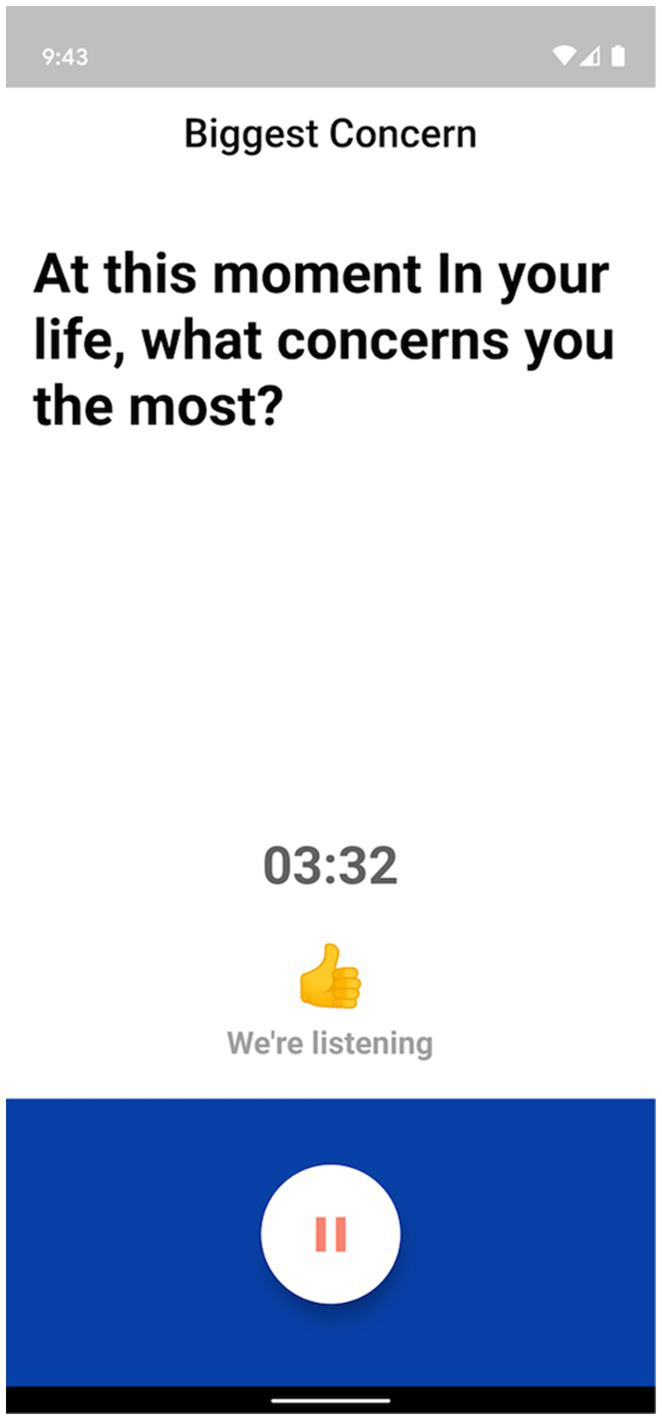
Question prompt for Ellipsis Health App participants.

The Ellipsis Health App empowers participants by allowing a diverse set of topics to talk about, including how they are performing self-care, the composition of their support structure, and how they feel about their current living situation (see Figure 1 for an example of in-app topics). Participants verbally answer one or more of these questions to provide the required 5 min of speech (Figure 2). The users’ de-identified data are transferred to cloud-based deep learning NLP models for binary classification results (i.e., presence or absence of depression and anxiety).

In previous research, Ellipsis Health introduced a General Population model based solely on semantic analysis of depression, built upon a younger age distribution with little overlap from the current study population. The model maintained performance when applied to senior populations such as the one examined in this study (Rutowski et al., 2021). Though Ellipsis Health has not reported on the portability of its improved transformer architecture to different populations, these previous results suggest portability across age groups without the need for significant retraining.

In summary, the Ellipsis Health App has a promising algorithm that detects depression and anxiety utilizing patients’ speech data. The patient-centered design attempts to improve upon existing mental health assessment tools and is likely to improve user engagement. Therefore, the primary aim of this study is to determine the feasibility of collecting weekly voice samples for semantic analysis of depression and anxiety. The secondary aim is to validate the portability and improvement upon the previous machine learning models to the study population, using the latest transformer methodologies.

## Materials and Methods

### Overview

Ellipsis Health (EH) is an early stage health technology company that is creating a scalable and objective way to measure the severity of depression and anxiety through artificial intelligence (AI) enabled analysis of the semantics and acoustics of patient speech.

In this study, EH partnered with Desert Oasis Healthcare (DOHC), a healthcare organization that provides medical care and wellness services for patients in Coachella Valley, California, and the surrounding desert communities of Riverside and San Bernardino counties in California. The aim of this partnership was to validate the screening of depression and anxiety using only the semantic analysis model of the EH solution. EH contracted the DOHC research department to carry out this study on a specified cohort of their patients.

The current study was a non-randomized prospective cohort study that took place between August 2018 and July 2020, per the approved Institutional Review Board protocol. Two groups of participants were recruited and enrolled, Group Positive (GP; participants with a documented history of depression within 24 months prior to study initiation) and Group Negative (GN; participants with no documented history of depression). This group segmentation of participants was created to demonstrate the feasibility and performance of the Ellipsis Health App and algorithms among people with or without a history of depression. Although anxiety was also measured in this study, there was no pre-study screening for this condition.

### Inclusion and Exclusion Criteria

Eligible participants were current DOHC patients meeting the following criteria: (a) between 18–80 years old, (b) completed Informed Consent form, and (c) had access to a smartphone (iOS or Android) as well as to the internet. Exclusion criteria specified participants with at least one of the following conditions: (a) Parkinson’s disease, (b) cerebrovascular accident (CVA) or head trauma with residual dysarthria within the prior 12 months, (c) amyotrophic lateral sclerosis (ALS), (d) congenital deafness, (e) severe cognitive deficits, (f) schizophrenia, (g) psychosis, or (h) employees of DOHC.

### Participants and Procedure

**Figure 3:**
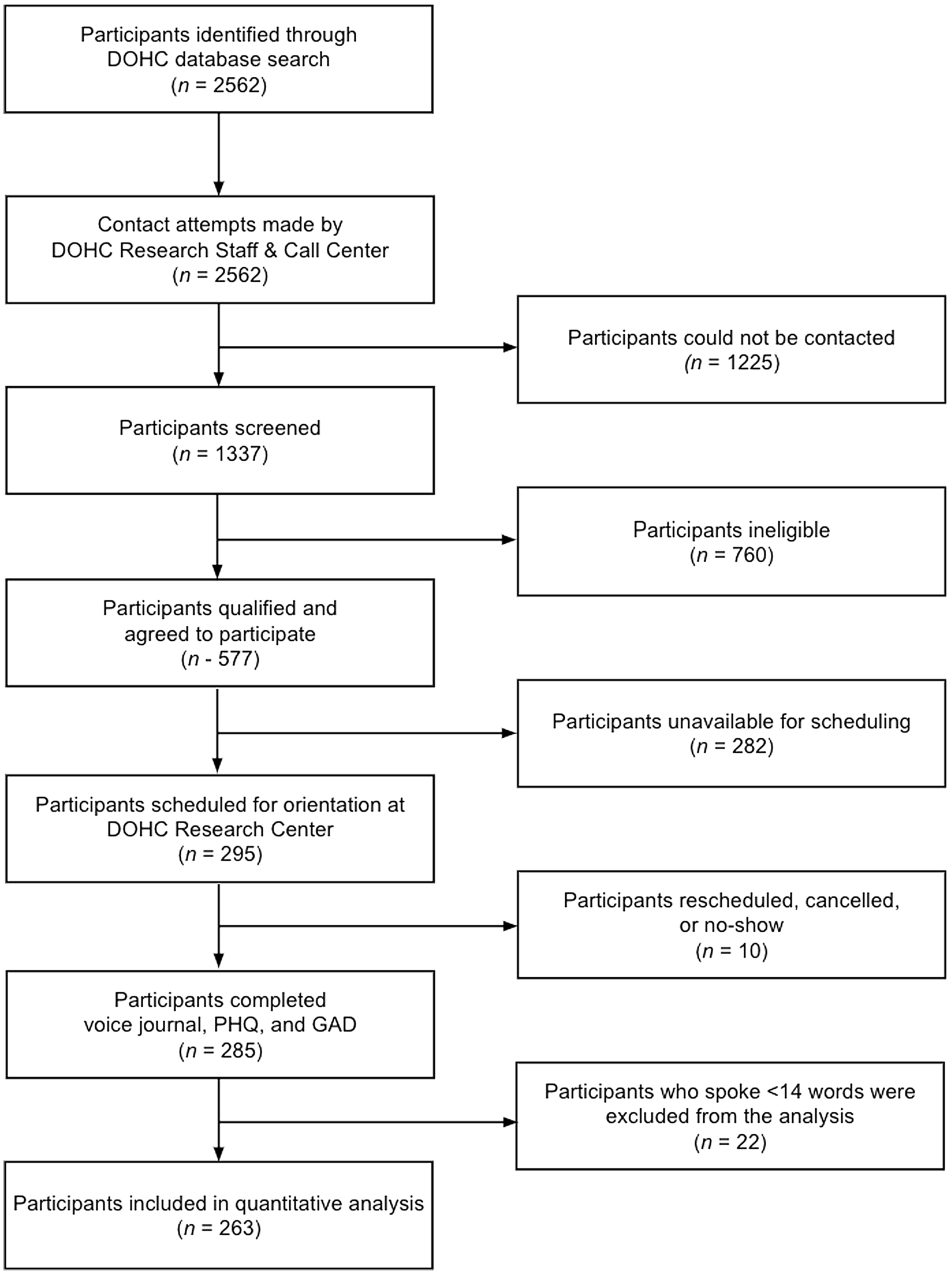
Overall Study Workflow.

A list of potential participants meeting the inclusion criteria was curated from the DOHC’s Electronic Health Record (see Figure 3). Recruitment was preceded by scripted phone contact by a DOHC Research Staff member or contracted agent *via* Altura, a private, for profit call center service that was contracted by Ellipsis Health.

Registration on day one consisted of filling out the participant’s name, Medical Record Number, and activation code (provided by research staff). Participants then received information about the trial, downloaded the Ellipsis Health App, were informed of the required six weekly 5-min voice samples, and completed the PHQ-8 and GAD-7 questionnaires. Subsequent sessions, consisting of a recorded voice sample and the two questionnaires, were completed at the participant’s home. The six repeated assessments per participant provided ample speech samples to validate algorithm performance in the study population, and helped train future models.

Prior to the first home session, participants received app notifications weekly at their preferred day and time to remind them to complete subsequent weekly sessions. Participants encountering technical difficulties were encouraged to contact the DOHC Research Center or Ellipsis Health clinical team by phone, email, or through the Ellipsis Health App. Out of a possible total of 1,578 speech assessment sessions, 188 were removed due to <14 words spoken per session (this being the threshold for model analysis; see below for details on automatic speech recognition) and 60 sessions were incomplete due to poor audio quality. The remaining 1,308 sessions were classified for the presence of depression and/or anxiety with the proprietary Ellipsis Health NLP machine learning algorithms.

Participants received monetary compensation for study completion *via* gift card, with each participant receiving no more than $50 for their effort and time. After the final (sixth) session, participants were asked to complete a survey about their experience. Participants were informed that they could use the App beyond the sixth session without compensation.

### Ellipsis Health App

Participants from GP and GN used the Ellipsis Health App for speech recordings. The Ellipsis Health App predicts the severity of depression and anxiety using two machine learning algorithms: one that analyzes the words spoken using natural language processing (NLP) engines and the other that analyzes the acoustic properties of the speech (Chlebek et al., 2020). For this study, only the NLP algorithm was utilized. The NLP algorithm relies on commoditized automatic speech recognition (ASR) technology, which parses the speech signal and separates out individual words. The ASR technology accounts for issues such as ambient noise, accents, and stuttering, while the NLP algorithm is trained to be equally reliable across speakers of different ages, races, dialectal regions, etc. (Rutowski et al., 2021). As with all technology that analyzes speech, disorders or conditions that affect the ability to produce spoken language may impact the results. No subjects included in this study reported any such speech disorders.

All data transfer, storage, and access operations were conducted in a manner that protected the privacy of the individual patients, per HIPAA guidelines. The algorithms returned a positive classification if the PHQ-8 or GAD-7 equivalent scores were greater than or equal to 10 (higher scores designate higher severity of depression and anxiety in both the PHQ-8 and GAD-7, respectively), a commonly used threshold cutoff for binary classification in clinical practice and in the mid-range of those reported in the literature (Spitzer et al., 2006; Manea et al., 2012).

### Measures

Each session finished with the participants completing both the PHQ-8 and GAD-7 through the Ellipsis Health App.

#### PHQ-8

The empirically validated PHQ-9 instrument for adults is a patient reported survey of nine questions regarding depression symptoms within the past 2 weeks. The last question was omitted in this study for liability reasons as it addresses suicidality. Patients rate symptoms on four levels of frequency.

#### GAD-7

The empirically validated GAD-7 instrument for adults is a patient reported survey of 7 questions regarding anxiety symptoms within the past 2 weeks. Patients rate symptoms on four levels of frequency.

#### Optional Post-study Survey

The optional Post-Study Survey was seven questions, completed after session 6 (Table 1). Two questions required free form responses, two questions were yes/no responses and three were based on a five-point Likert scale.

**Table 1:** Study completion survey.

Question			
1	Please rate how easy the study was for you?		
	1 – Very Easy	3 – Okay	5 – Very Difficult
2	If you encountered any difficulty over the six sessions performing the study, please describe them.		
3	Would you do a single session once per year while waiting at your doctor’s office? Please answer yes or no.		
4	Did you feel the study felt repetitive after several sessions?		
	1 – Very Repetitive	3 – It was fine.	5 – Look forward to doing it
5	Did you feel the compensation was appropriate for your participation?		
	1 – Not enough	3 – About right	5 – Too much
6	Would you be interested in improving behavioral health today by partnering with Ellipsis Health? Your interaction with new products and feedback regarding emotional assessment is extremely valuable.		
7	Please share any other comments and suggestions about participating in this study?		

#### Ellipsis Health App Feasibility

For each participant, the percentage statistic of the number of completed assessments over six possible sessions was calculated and the App’s feasibility was thought to be achieved if a majority of subjects met or exceeded the research protocol specified six sessions.

#### Ellipsis Health App Portability and Improvement

Portability is defined here as the ability of a machine learning model to perform on a new population within 10% of its performance on the population on which it was trained, where performance is expressed in terms of AUC. Improvement is defined here as a positive change in AUC when comparing the former machine learning model that was based upon long short-term memory (LSTM) techniques to the newer Transformer model.

### Statistical Analysis

Descriptive statistics included study participants’ race, age, gender, and the number of sessions completed. The statistical analysis of data was carried out using various python statistical packages, including statsmodel, pingouin, scipy, and pandas. Only statistically significant values of *p* (*p* ≤ 0.05) are reported. Confidence intervals (CI) of 95% are also reported when appropriate, for example, for Area Under the Curve (AUC).

GP and GN differences in categorical variables were calculated with Chi-squared tests. Pairwise differences between categorical and continuous variables were carried out using *t*-tests. When more than two categorical variables were involved, ANOVA or Welch-ANOVA was carried out depending on the satisfaction of variance criteria of categorical variables. If ANOVA or Welch-ANOVA identified statistically significant differences, pairwise *t*-tests were carried out to calculate the value of *p*. In certain cases, continuous variables were binned to facilitate calculation using the Spearman or Pearson correlation tests (Tables 2, 3, 4 for actual values of *p*).

**Table 2:** Demographic characteristics of Desert Oasis Healthcare study participants for Group Positive and Group Negative Chi-square and *t*-tests.

Variable	Value of *p*	Statistical test
**Race** (*n* = 177)^1^		
White, non-white	0.66	Chi-square
**Gender** (*n* = 260)		
Male, Female	0.46	Chi-square
**Age** (*n* = 260)		
<60, 60–70, >70	0.02	Chi-square
Mean	0.01	*T*-test
**Number of sessions completed** (usage frequency; *n* = 263)		
<6, 6, >6	0.11	Chi-square
Mean	0.87	*T*-test

1 *Removed responses for those who declined to answer and/or had erroneous race categories*.

**Table 3:** Demographics and session duration/frequency – ANOVA, Welch’s ANOVA and Chi-squared tests with initial PHQ/GAD scores and survey answers in Group Positive participants.

Categories	PHQ: mean scores (value of *p*, ANOVA)	GAD: mean scores (value of *p*, ANOVA)	Mean recording duration (value of *p*, ANOVA)	Average number of sessions (value of *p*, ANOVA)	Survey question #1: how easy mean (value of *p*, ANOVA)	Survey question #3: annual survey at doctor’s office (value of *p*, Chi-square)	Survey question #4: How repetitive mean (value of *p*, ANOVA)	Survey question #5: compensation mean (value of *p*, ANOVA)
**Gender** (*n* = 238)								
Male Female	0.81	0.85	0.71	0.47	0.019^1^	0.79	0.79^1^	0.66^1^
**Age** (*n* = 238)								
<60 60–70 >70	0.004^1^	<0.00^1^	0.24	0.49	0.75	0.12	0.31	0.57
**Race** (*n* = 155)								
White Non-white	0.69	0.42	0.98	0.20	–	–	–	–
**Number of sessions completed** (*n* = 240)								
<6, 6, >6	0.028	0.11^1^	0.67	–	0.23	0.87	0.69	0.35

1 *Welch’s ANOVA, (−) No results shown due to small sample size*.

**Table 4:** Age, number of completed sessions and *post-hoc* Gender Pairwise *t*-test on statistically significant differences determined by ANOVA with initial PHQ/GAD scores and survey questions of Group Positive participants.

Category 1	Category 2	Value of *p* (PHQ mean)	Value of *p* (GAD mean)
**Age** (*n* = 238)			
<60	60–70	0.006	<0.001
60–70	<70	0.34	0.10
<60	>70	0.001	<0.001
**Number of Sessions Completed** (*n* = 240)	
<6 sessions	6 sessions	0.48	–
6 sessions	>6 sessions	0.16	–
<6 sessions	>6 sessions	0.036	–
**Gender** (*n* = 238)		Value of *p* (survey question #1: how easy)	
Male	Female	0.019	

In addition to primary analyses, we also conducted a set of exploratory analyses to evaluate the model performance over all thresholds. We used AUC as our metric, where 0.5 AUC is equivalent to chance. This measures the ability of an algorithm to distinguish between a patient with or without a condition (in this case, depression and anxiety). The CI of AUC is calculated using DeLong’s test (DeLong et al., 1988).

The Equal Error Rate (EER) point is the point on the AUC curve where the sensitivity and specificity of the assessment tool are equal. For GP, the EER is at 75.4% for PHQ-8 and 75.1% for GAD-7. Thus, we used 75% specificity as the “set point” for calculations of the AUC curve. We also included Positive Predictive Value (PPV), the likelihood that a positive test indicates the individual has the condition, and Negative Predictive Value (NPV), the likelihood that the negative test indicates the individual does not have the condition.

## Results

### Study Population

The study included 263 DOHC subjects (GN *n* = 23 and GP *n* = 240). Of these, 3 declined to report their age or gender and 54 declined to state their race. Subsequent results are based only on the demographics that were reported. Ages ranged from 22 to 73, with a mean age of 60 (SD = 9.8) in GP and 56 (SD = 12.5) in GN. GP had 61% female subjects and GN had 50% female subjects (Table 5). The majority of the subjects in both groups were Caucasian and did the required 6 (or more) sessions. The protocol completion rate in this study (defined as completing 6 or more sessions) was 14/23 (61%) in GN and 147/240 (61%) in GP. 27% of GP and 9% of GN used the Ellipsis Health App beyond 6 sessions without further compensation. Out of a total of 1,308 sessions in both groups, 478 were longer than the required 5 min. GP and GN were compared by race, gender, age and number of sessions completed. The only significant difference was in mean age, where 50% of GN subjects were under 60 years compared to 24% in the GP (Table 2). While the majority of GP subjects used the app at least six times, they spoke for less than 5 min on average per session (250.5 s, SD = 60.6; Table 6). The average duration of the first recording was 4 min, and the average of the shortest session recording was approximately 3 min. Since the GN group size was ~10% of the GP group, subsequent results reported in this manuscript were calculated based solely on GP, unless otherwise noted, as this disparity in size is inadequate for more in-depth statistical analyses.

**Table 5:** Demographic characteristics of Desert Oasis Healthcare study participants for Group Positive (those with a history of depression) and Group Negative (those without).

Variable	Group Positive	Group Negative
**Race** (*n* = 247)		
Black	8	1
White	151	10
Other	6	1
Declined to answer	60	10
**Gender^1^** (*n* = 260)		
Male	94	11
Female	144	11
**Age^1^** (*n* = 260)		
<60	56	11
60–70	125	9
>70	57	2
Mean (SD)	64 (9.8)	56 (12.5)^2^
**Number of sessions completed** (*n* = 263)		
<6	93	9
6	83	12
>6	64	2
Mean (SD)	5.0 (2.4)	4.9 (1.8)
**Total subjects per group**	**240**	**23**

1 *Group sums less than the total amount per group reflect cases in which demographic data was not self-reported*.

2 *T-test reveals significance, p ≤ 0.05*.

**Table 6:** Demographics, initial PHQ/GAD scores, and session duration/average number for Group Positive participants.

Categories	Number (%)	PHQ: mean scores (SD)	GAD: Mean scores (SD)	Mean recording duration (SD)^3^	Average number of sessions (SD)
**All Group Positive**	240 (100%)	7.5 (5.1)	5.8 (4.8)	245.5 (106.2)	5.0 (1.0)
**Number of sessions completed** (usage frequency; *n* = 240)					
<6	93 (38.8%)	8.5 (−)^1^^,^^2^	6.4 (−)^1^	239.0 (−)^1^	–
6	83 (34.6%)	7.3 (2.5)^2^	5.9 (2.2)	253.2 (62.0)	–
>6 (Mean = 7.4,SD = 1.2)	64 (26.7%)	6.2 (2.3)^2^	4.8 (2.0)	244.7 (58.2)	–
**Gender** (*n* = 238)					
Male	94 (39.2%)	7.4 (2.2)	5.8 (1.8)	242.3 (60.2)	4.8 (2.6)
Female	144 (60.0%)	7.6 (2.6)	5.9 (2.3)	247.4 (60.5)	5.1 (2.6)
**Age** (n = 238, Mean = 60.4, SD = 9.8, range = (22–73)					
<60	56 (23.3%)^3^	9.8 (2.5)^4^	8.9 (2.4)^4^	256.0 (54.5)	5.2 (2.0)
60–70	125(52.1%)^3^	7.1 (2.4)^4^	5.2 (2.2)^4^	249.6 (62.8)	5.0 (2.6)
>70	57 (23.8%)^3^	6.3 (2.5)^4^	4.2 (1.7)^4^	225.5 (61.3)	4.7 (2.3)
**Race** (*n* = 165)^5^					
White	151 (62.9%)^3^	7.3 (2.4)	5.6 (2.1)	253.7 (57.9)	5.1 (2.3)
Non-white	14 (5.8%)^3^	7.9 (2.4)	6.6 (2.8)	252.8 (44.6)	4.3 (3.0)

1 *SD (Standard Deviation) not calculated for groups with fewer than 6 total sessions; post hoc tests showed non-significant differences unless otherwise specified*.

2 *ANOVA reveals significant difference, p ≤ 0.05*.

3 *Group sums less than the total amount per group reflect cases in which demographic data was not self-reported*.

4 *Welch ANOVA reveals significant difference, p ≤ 0.05*.

5 *Removed responses for those who declined to answer and/or had erroneous race categories*.

### Bivariate Association Between Demographic Statistics and Initial Depression and Anxiety Scores

Demographics and total sessions completed and their initial PHQ-8, GAD-7 and session duration in GP are shown in Table 6. Correlations between most demographics versus initial PHQ-8 and initial GAD-7 scores were not statistically significant. Increasing age was statistically correlated with lower PHQ/GAD scores. Increasing number of sessions completed was statistically correlated with lower initial PHQ-8 scores. Internal consistency of PHQ-8 scores among study participants was satisfactory, Cronbach’s alpha = 0.90 ([CI 0.90–0.91]). Internal consistency of GAD-7 scores among study participants was also satisfactory, Cronbach’s alpha = 0.92 (CI [0.91–0.93]).

### Survey Results

Demographics and number of sessions completed grouped by <6, 6 or > 6 in relation to survey questions #1,3,4,5 are shown in Table 7. Male gender correlated with rating the app easier to use, but no other demographic factors measured, or the number of sessions completed was statistically correlated with answers to the selected survey questions.

**Table 7:** Demographics and survey answers (see Table 1) in Group Positive participants (*N* = 103).

Categories	Survey Question #1: How Easy Mean (SD)^1^	Survey Question #3: Annual survey at doctor’s office (%)^2^	Survey Question #4: How Repetitive Mean (SD)^1^	Survey Question #5: Compensation Mean (SD)^1^
**Group Positive set**	2.1 (1.3)	60.2%	2.2 (1.2)	2.8 (0.8)
**Gender**				
Male	1.8 (0.7)^3^	63.1%	2.1 (1.1)	2.8 (0.7)
Female	2.3 (0.9)^3^	58.5%	2.2 (1.3)	2.8 (0.9)
**Age** [Mean = 64.0, SD = 9.8, range = (22–73)]				
<60	2.3 (1.2)	45.5%	1.9 (1.2)	2.6 (0.7)
60–70	2.0 (1.2)	69.1%	2.3 (1.2)	2.9 (0.8)
>70	2.2 (1.4)	53.8%	2.0 (1.4)	2.8 (0.8)
**Race**				
White	2.2 (1.3)	56.5%	3.1 (1.2)	2.9 (0.8)
Non-white^4^	–	–	–	–
**Number of sessions completed** (mean, SD)				
<6 (Mean = 2.4, SD = 1.4)	2.8 (1.3)	50.0%	2.5 (1.5)	2.3 (1.0)
6	2.0 (1.2)	61.1%	2.1 (1.1)	2.8 (0.7)
>6 (Mean = 7.4, SD = 1.2)	2.2 (1.3)	60.5%	2.2 (1.4)	2.8 (0.9)

1 *Mean response from Likert scale of 1–5*.

2 *% ‘Yes’ response*.

3 *ANOVA reveals significant difference, p ≤ 0.05*.

4 *No results given due to small sample size.*

### Summary of Comparisons

Tables 2, 3, 4 show results for all ANOVA, *post-hoc* Chi-squared and pairwise *t*-tests performed on selected demographics and number of completed sessions grouped by <6, 6 or > 6 compared to initial PHQ/GAD, recording duration and average number of completed sessions.

### Receiver Operating Curve

**Figure 4:**
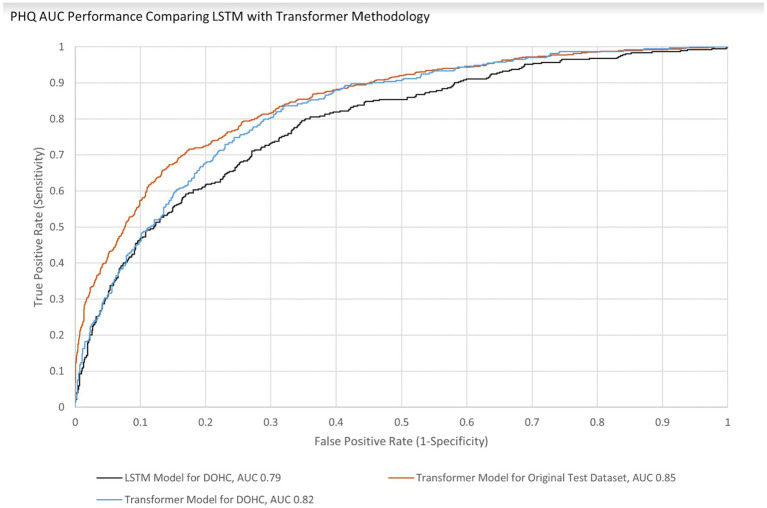
PHQ AUC Performance Comparing LSTM with Transformer Methodology for Both GP and GN.

**Figure 5:**
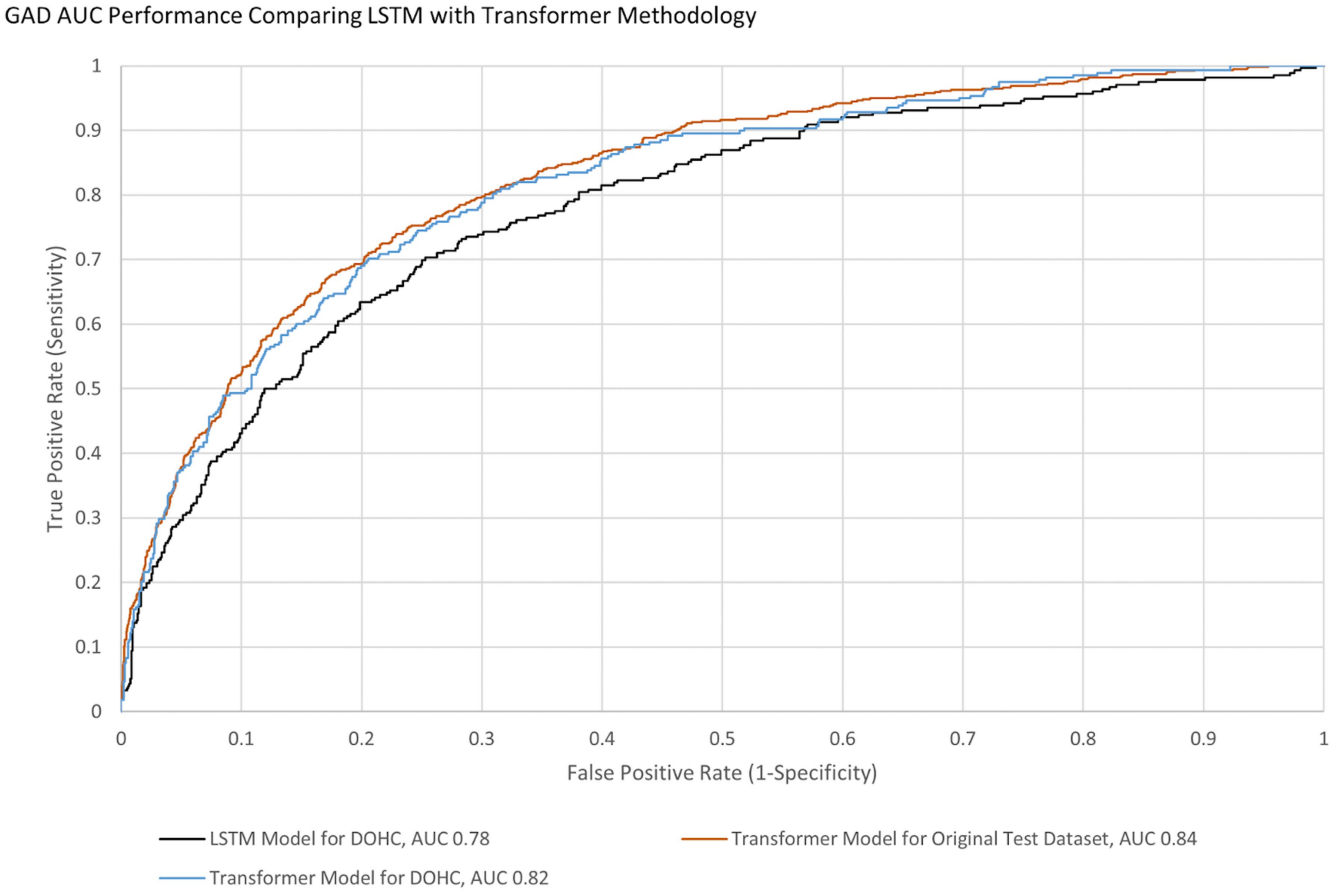
GAD AUC Performance Comparing LSTM with Transformer Methodology for Both GP and GN.

AUCs were initially calculated for the first session only to simplify analysis. The AUC performance of the transformer model predicting the first session PHQ-8 of both groups (GP and GN) combined was 0.82 [95% CI (0.797, 0.846)] with an EER of 0.75 and PPV of 0.54 and NPV of 0.88, while for the GP group only it was 0.83 [95% CI (0.801 0.85)] with an EER of 0.75 and PPV of 0.54 and NPV of 0.89 (Table 8). This is within 10% of an AUC of 0.85 for the transformer model performance on the original test dataset, indicating portability. With GAD-7, the AUC of the Transformer model for both groups were 0.82 [95% CI (0.790, 0.846)] with an EER of 0.75 and PPV of 0.45 and NPV of 0.91 and for the GP group only the AUC was 0.83 [95% CI (0.80 0.86)] with an EER of 0.75, PPV 0.44, and NPV 0.92. The transformer model performance on the original test dataset for GAD-7 is 0.84, which is within 10% of the performance on this study’s population, also indicating portability. The AUC performance of the older LSTM model on both groups (GP and GN) combined for the first session PHQ-8 is 0.79 compared to the transformer model AUC of 0.82, indicating a 4% improvement (Figure 4). The AUC performance of the older LSTM model on both groups combined for the first session GAD-7 is 0.78 compared to the transformer model AUC of 0.82, indicating a 5% improvement (Figure 5).

**Table 8:** Semantic algorithm performance for initial PHQ and GAD scores.

Measure	AUC^1^ (95% CI)	EER^2^	PPV^3^	NPV^4^
**All subjects** (*n* = 263)				
Initial PHQ	0.82 (0.80, 0.85)	0.75	0.54	0.88
Initial GAD	0.82 (0.79, 0.85)	0.75	0.45	0.91
**Group Positive subjects** (*n* = 240)				
Initial PHQ	0.83 (0.80, 0.85)	0.75	0.54	0.89
Initial GAD	0.83 (0.80, 0.86)	0.75	0.44	0.92

1 *AUC (Area Under Curve)*.

2 *EER (Equal Error Rate)*.

3 *PPV (Positive Predictive Value)*.

4 *NPV (Negative Predictive Value)*.

**Figure 6:**
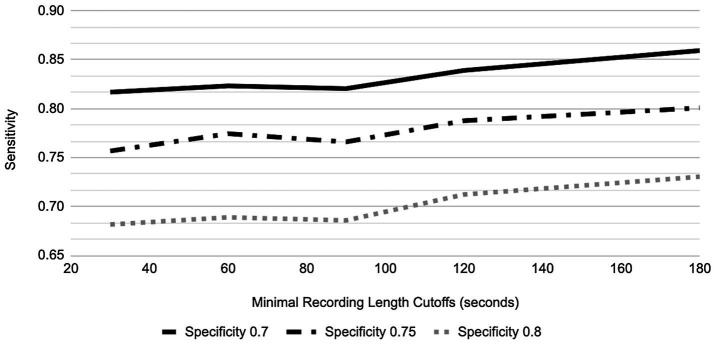
Sensitivity vs. Specificity in relation to recording length cutoffs for PHQ-8.

**Figure 7:**
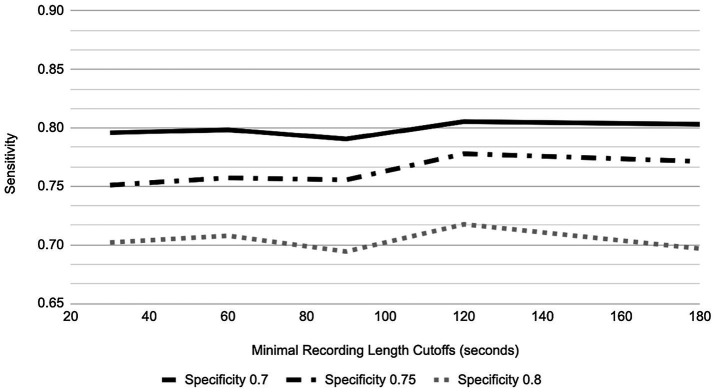
Sensitivity vs. Specificity in relation to recording length cutoffs for GAD-7.

In order to provide a generic AUC result more representative of all 6 sessions and not influenced by the subject’s tendency to record less or more than the protocol defined 5 min, AUC performance in the GP for different speaking lengths was explored (Figures 6, 7). For 80% of participants, their shortest session was <5 min; in fact, the mean of the shortest session within GP participants was 189 s and 15% of GP participants have their longest session <3 min. By placing the cutoff at 3 min, we include the AUC performance from as many participants as possible while not losing a majority of data, retaining 67.8% of recordings and 85.0% of participants.

For the three different designated specificity levels of 0.70, 0.75, and 0.80, the AUC performance (as represented by varying sensitivity) for PHQ-8 prediction has an increasing trend as speaking length increases, from the shortest speaking length of ~20 s to 180 s (Figure 6). However, for GAD-7 there was minimal change in performance at the specificity level of 0.80, while the two lower designated specificity levels showed a trend toward improved sensitivity with speaking length (Figure 7).

## Discussion

The primary aim of this research was to determine the feasibility of voice assessment for binary classification detection of depression and anxiety as evidenced by the proportion of subjects who completed 6 or more sessions (6 being the number of sessions defined in the protocol), end-of-study survey feedback and other relevant app usage statistics. The secondary aim was to document the portability and performance of the machine learning models for depression and anxiety among speakers of various age groups using the newer transformer methodology.

Low engagement rates within mental health apps are a common feasibility challenge (Ng et al., 2019). The 61% completion rate of the protocol for participants in this study is less than participants in general clinical research (Center for Information and Study on Clinical Research Participation, 2019), but is similar to reported attrition rates in digital programs for mental health (Linardon and Fuller-Tyszkiewicz, 2020). However, unexpectedly, 27% of GP and 9% of GN participants performed more sessions than required. Due to the smaller sample size of GN, their percentage may be underestimated. Interestingly, increasing age correlated with lower initial PHQ-8 and GAD-7 scores, which is consistent with previous findings (Girgus et al., 2017). Lower PHQ-8 scores also correlated with an increased number of completed sessions, boding well for an app of this type in this more senior population. In fact, 36% of subjects spoke for more than the required amount of time, revealing that engagement with these subjects is a product of more than monetary compensation alone. This group of “overachievers” deserves further study to understand the cathartic response of verbalizing personal issues in this scenario. It is possible that certain personality archetypes (extroversion) respond favorably to speech-based assessments, and empowering subjects to choose questions for a personalized approach provides a positive mental health app experience.

Answers to the post-study survey [completed by 109/245 (44%) of the GP and 15/25 (60%) of the GN] were not statistically different, with the exception of male gender correlating with a rating of the app being easier to use. A majority of subjects reported that using the Ellipsis Health App was easy, and that they would be willing to complete similar assessments with their provider. This suggests that the Ellipsis Health App is an acceptable digital tool that can be used for annual screening with a provider. However, many participants found the Ellipsis Health App to be repetitive and thought the compensation was nominal at best. This is likely due to the App being primarily designed as a data collection and proof of concept tool rather than a commercial product. Since this study, the App has gone through multiple iterations to improve the user interface and user experience informed by similar surveys and formal user testing exercises with much improved feedback. These changes to the Ellipsis App include improved user engagement by providing an efficient onboarding process, reminders to provide a voice sample *via* push notifications, and a simplified workflow for the user to select the question they would like to answer.

Smartphone applications that make clinically valid assessments supported by published results in peer reviewed literature are in the minority of what is currently available in app stores (Krebs and Duncan, 2015; Firth et al., 2017). Usage statistics and survey results from this study taken together indicate that utilizing regular voice recordings of users answering questions from a smartphone app to analyze their levels of anxiety and depression is feasible. Ellipsis Health has previously published results of semantic (Rutowski et al., 2019, 2020) and acoustic (Harati et al., 2021) analysis of speech to detect depression and anxiety using models trained, to the best of our knowledge, with the largest database reported in the literature (Rutowski et al., 2019). We have also previously reported this algorithm performance is maintained (i.e., is portable) when applied to the current study population using long short-term memory (LSTM) models (Rutowski et al., 2020). The newer transformer methodology was also portable (performance was within 10% when comparing AUCs of the original training dataset and this study’s dataset). Additionally, this methodology also demonstrated an improved AUC of 4–5% when compared to the original training dataset population (Figures 4, 5) thereby increasing the prediction accuracy for depression and anxiety. Due to the accelerated evolution of ML techniques as well as the ever-increasing accumulation of labeled data, these results will continue this improvement trend. A framework is being developed by the FDA to account for continual algorithm development as described in “Artificial Intelligence/Machine Learning (AI/ML)-Based Software as a Medical Device (SaMD) Action Plan” which is pending formal regulation [United States Food and Drug Administration/Center for Devices and Radiological Health (FDA), 2021]. This will accelerate the benefits to all aspects of healthcare, leading to improved outcomes and decreased costs (Luppa et al., 2012; Melek et al., 2014).

### Limitations

Study limitations addressed by future research will make results more widely applicable to the clinical community. GN stipulates no documented history of depression only; future studies should specify no history of depression and/or anxiety as a comparison group. Though compensation as in this study may not reflect real-world use, incentives to influence subject behaviors are not uncommon in the clinical practice (Soliño-Fernandez et al., 2019; Vlaev et al., 2019). Our training dataset contains mixed ethnicities, but is mostly Caucasian (Rutowski et al., 2021), which does not reflect the country’s population. Further studies should address this discrepancy, especially since there are indications that treatment responses are similar (Lesser et al., 2011). Our study required smartphone usage, something older populations are not as comfortable with Connolly et al. (2018); however, as digital native and facile generations mature, this limitation will become trivial. In addition to technological literacy, the use of this app also requires access to smart devices and a reliable internet connection. Though these conditions are becoming increasingly ubiquitous, there are patient communities that cannot be expected to have access to devices and the internet, and therefore would not benefit as much from a mental health app such as this. Depression and anxiety occur throughout life, although with less prevalence in seniors (Girgus et al., 2017), so this app must appeal to a wider audience. Future studies exploring key age-specific engagement features for voice assessment of depression and anxiety need to be performed, and Ellipsis Health has these in process. Binary classification is useful in some cases, but more research on regression and multi-class classification models (i.e., none, mild, moderate, severe), which Ellipsis Health plans to publish, is necessary for other critical use cases. Lastly, the results reported in this study utilized only semantic analysis of speech. Acoustic analysis is an important component of speech analytics (Melamed and Gilbert, 2011) and Ellipsis Health plans to publish more results on acoustic algorithms in the future.

## Conclusion

This study aimed to determine the feasibility of assessing the presence of clinical depression and anxiety *via* commonly used thresholds (PHQ-8 and GAD-7 scores of 10 or greater) through a smartphone app that semantically analyzes speech through machine learning algorithms. The secondary aim was to show portability and improvement of the newer algorithms to this study population. Many participants talked to the Ellipsis Health App longer than required during at least one of their weekly sessions, and many completed more than the required six sessions even without extra compensation. A post-study survey showed general acceptance of the app. While ML performance on this study population has previously been reported, a newer methodology (Transformers) shows portability to a population that is generally older than the training population and improved performance compared to an older methodology (LSTM). Using free form speech to assess the presence of depression and anxiety is a viable technique in a generally senior population and may even be therapeutic for certain individuals.

## Data Availability Statement

The datasets presented in this article are not readily available because many that support findings for this study are proprietary. Requests to access the datasets should be directed to MA, mike@ellipsishealth.com.

## Ethics Statement

This study involving human participants was reviewed and approved by Advarra. The patients/participants provided their written informed consent for participation.

## Author Contributions

DL designed the study and authored and submitted the study design for IRB approval. TN revised the IRB proposal, as well as contributing to data collection and serving as the technical support coordinator for the study. TR, YL, AH, ES, and PC all contributed to creating the machine learning models that are evaluated here. TR also calculated model results. YL also performed all the statistical tests reported in the paper. MA supervised and coordinated all authors throughout the process of receiving IRB approval for this study and performed final review and revisions of the manuscript. All authors contributed to writing and revising the manuscript.

## Funding

All of the authors are employees of Ellipsis Health, who is the sole funder of this study.

## Conflict of Interest

All authors were employed by the company Ellipsis Health Inc. The authors declare that this study received funding from Ellipsis Health Inc. Ellipsis Health Inc. had the following involvement in the study: study design, collection, analysis, interpretation of data, the writing of this article and the decision to submit it for publication.

## Publisher’s Note

All claims expressed in this article are solely those of the authors and do not necessarily represent those of their affiliated organizations, or those of the publisher, the editors and the reviewers. Any product that may be evaluated in this article, or claim that may be made by its manufacturer, is not guaranteed or endorsed by the publisher.

## Abbreviations

AI: Artificial Intelligence
ALS: Amyotrophic Lateral Sclerosis
ANOVA: Analysis of Variance
AUC: Area Under Curve
ASR: Automatic Speech Recognition
CVA: Cerebral Vascular Accident
CI: Confidence Interval
DOHC: Desert Oasis Healthcare
EH: Ellipsis Health
EER: Equal Error Rate
FDA: United States Food and Drug Administration
GAD: Generalized Anxiety Disorder
GAD-7: Generalized Anxiety Disorder (7-item questionnaire)
GN: Group Negative
GP: Group Positive
iOS: iPhone OS
HIPAA: The Health Insurance Portability and Accountability Act
LSTM: Long Short-Term Memory
ML: Machine Learning
NLP: Natural Language Processing
NPV: Negative Predictive Value
PHQ: Patient Health Questionnaire
PHQ-8: Patient Health Questionnaire (eight—item questionnaire)
PHQ-9: Patient Health Questionnaire (nine-item questionnaire)
PPV: Positive Predictive Value
ROC: Receiver Operating Curve
SD: Standard Deviation
SaMD: Software as a Medical Device
Welch’s ANOVA: Welch’s Analysis of Variance
